# Mapping current status and emerging trends in NETosis: A bibliometric study

**DOI:** 10.1097/MD.0000000000033806

**Published:** 2023-05-26

**Authors:** Hongqin Wang, Xiaolin Liu, Zijun Jia, Li Liu, Yifei Qi, Qingbing Zhou, Fengqin Xu, Ying Zhang

**Affiliations:** a Institute of Geriatric, Xiyuan Hospital, China Academy of Chinese Medical Sciences, Beijing, China; b Beijing University of Chinese Medicine, Beijing, China.

**Keywords:** autoimmune, bibliometric, cancer, coronavirus disease 2019, neutrophil extracellular traps

## Abstract

NETosis is a critical innate immune mechanism of neutrophils that contributes to the accelerated progression of autoimmune diseases, thrombosis, cancer, and coronavirus disease 2019 (COVID-19). This study qualitatively and quantitatively analyzed the relevant literature by bibliometric methods in order to provide a more comprehensive and objective view of the knowledge dynamics in the field.

**Methods:**

The literature on NETosis was downloaded from the Web of Science Core Collection, analyzed with VOSviewer, CiteSpace, and Microsoft for co-authorship, co-occurrence, and co-citation analysis.

**Results:**

In the field of NETosis, the United States was the most influential countries. Harvard University was the most active institutions. Mariana J. Kaplan and Brinkmann V were, respectively, the most prolific and most co-cited authors. Frontiers in Immunology, Journal of Immunology, Plos One, Blood, Science, Journal of Cell Biology, and Nature Medicine were the most influential journals. The top 15 keywords are associated with immunological and NETosis formation mechanisms. The keywords with the strongest burst detection were mainly related to COVID-19 (coronavirus, ACE2, SARS coronavirus, cytokine storm, pneumonia, neutrophil to lymphocyte ratio), and cancer (circulating tumor cell).

**Conclusion:**

Research on NETosis is currently booming. The mechanism of NETosis and its role in innate immunity, autoimmune diseases, especially systemic lupus erythematosus and rheumatoid arthritis, and thrombosis are the focus of research in the field of NETosis. A future study will concentrate on the function of NETosis in COVID-19 and recurrent metastasis of cancer.

## 1. Introduction

NETosis is a program that activates neutrophils to release neutrophil extracellular traps (NETs), which are composed of modified chromatin and bactericidal proteins from granules and cytoplasm.^[1]^ Depending on whether the neutrophil plasma membrane is ruptured and whether it continues to perform phagocytosis, NETosis can be classified into suicidal NETosis and vital NETosis.^[2]^ Suicidal NETosis is a programmed cell death with neutrophils releasing NETs when the cytoplasmic membrane ruptures, while vital NETosis extrudes NETs in a vesicular outgrowth manner, in which the granulocyte surface membrane is intact, still contains granules, and is phagocytic.^[3,4]^ NETosis can be induced by bacteria, bacterial toxins, fungi, parasites, phorbol myristate acetate (PMA), lipopolysaccharide, immune complexes, activated platelets, cholesterol crystals, antibodies, interleukin-1β, tumor necrosis factor-α and so on.^[5,6]^ Distinct stimuli can induce different types of NETosis; for example, PMA often induces suicidal NETosis, while microbes and activated platelets often induce vital NETosis. Due to the complexity of NETosis, the Nomenclature Committee on Cell Death has recommended that “NETosis” should not be used in the absence of definite observation of neutrophil death.^[7]^ Sebastian Boeltz and his colleagues^[8]^ have recommended using “NETosis” only in cases of apparent neutrophil death, and the term “NETs formation” is recommended in all other cases. Innate immunity is the most prevalent, rapid-acting, and critical type of immunity, it serves as the host’s first line of defense against microbial infection.^[9,10]^ The antigen presentation function and cytokine production of innate immune cells are prerequisites for adaptive immune responses.^[9,10]^ Neutrophils, as the effector cells of innate immunity, play a crucial role in combating infections. Additionally, NETosis, a process in which neutrophils release NETs, is essential in innate immunity, NETs can bind and kill pathogens, but excess NETs are involved in the development and progression of autoimmune diseases, thrombosis, cancer, coronavirus disease 2019 (COVID-19), cardiovascular disease, diabetes, neurological disorders, acute lung injury, and other diseases.^[11,12]^

Bibliometrics has become an important tool in the research world for judging the impact or value of research.^[13]^ It enables us to compare not only the contributions of various countries, institutions, journals, and scholars but also to realize the dynamic development of scientific research through quantitative measurement of the relationships and clustering of studies.^[14,15]^

Thus far, there is little bibliometrics for NETosis; hence, CiteSpace and VOSviewer were applied in this study to perform analysis and visualization of the results of NETosis-related literature as a knowledge base in this field and to explore its research hotspots and frontiers for further research.

## 2. Materials and methods

### 2.1. Data source and search strategy

Science Citation Index Expanded from the Web of Science core collection (WoSCC) database was utilized for our article searches. Because WoSCC is recognized as a collection of high-quality, peer-reviewed academic literature, that 1 was chosen as the database, and the science citation index expanded includes the world’s leading academic journals in the natural sciences, clinical medicine, and other fields by strict selection criteria, helping to ensure the quality of the research papers. Data were collected during a single day on May 26, 2022, to prevent deviations caused by daily database updates. The search criteria were set as follows: TS = (“NETosis” or “Neutrophil Extracellular trap*s”), language = English; document types = “Articles” or “Review Articles.” Ultimately, a total of 4873 articles were retrieved, including 3384 articles and 1489 reviews. The final retrieved results were exported from the WoSCC as plain text files with full record and citation information in the name download_xxx, and the data files were later imported into bibliometric software for de-duplication and analysis, 3 duplicates were removed, and the final 4870 documents were included in the analysis.

### 2.2. Data analysis

CiteSpace 5.8.R3 and VOSviewer with their parameters set to default values, as well as Microsoft Excel 2019, were used as analytical tools. This article does not involve any human or animal subjects, therefore informed consent is not applicable.

Using Microsoft Excel to plot tables and chart trends in publications and citations.

CiteSpace 5.8.R3 was used to produce visualization maps to analyze the co-authorship, co-citation, and co-occurrence of authors, keywords, and references. CiteSpace is a visual and analytical citation software for analyzing potential knowledge in scientific research.^[16]^ CiteSpace’s analysis mapping consists mainly of nodes and lines. Nodes indicate research items and are usually composed of different colored annual rings, where the annual rings in the nodes indicate the number of items appearing in different years. The width of the annual rings is proportional to the frequency of items that the rings represent. The node size corresponds to how frequently it appears. Moreover, red and purple are highlighted to show the special properties of the nodes.^[15]^ A red ring signifies the detection of a citation burst in the correlative time slice. A purple ring is an additional ring with thickness proportional to its centrality value when the node’s betweenness centrality (BC) is > 0.1.^[17]^ BC is a measure of the percentage of shortest paths for a given node in the network to which it belongs, and nodes with high BC are the hubs that bridge distinct clusters.^[18]^ Lines represent cooperation, co-citation, or co-occurrence relationships among nodes. The thickness and frequency of the lines indicate the strength of these correlations of the connections between the nodes, and the color of the lines indicates the chronological order of occurrence of the item.^[19,20]^

Using VOSviewer to analyze co-cited journals and create density maps. VOSviewer is a computer program used to create and view bibliometric maps in an in an easy-to-understand format.^[21]^ In this view, research items are represented by circles and labels whose size is in proportion to the importance of the item, with each item’s circle shown in the item’s color.^[21]^

## 3. Results

### 3.1. Annual publications and citations

**Figure 1.**
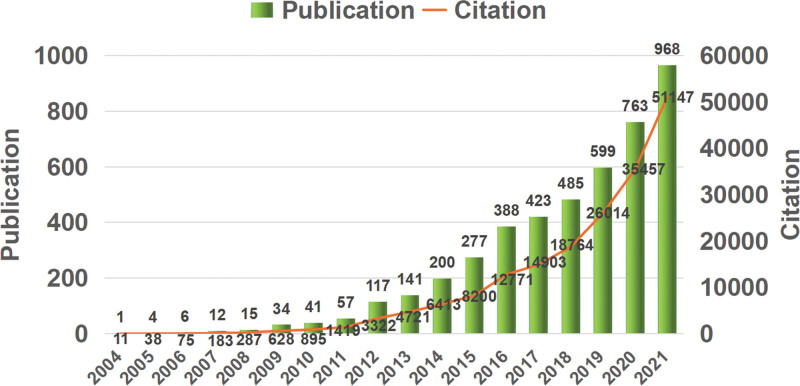
The annual frequency of publications and citations on NETosis (The number of publications and citations is represented by bar graphs and line graphs, respectively, with the number.

As shown in Figure 1, NETosis-related papers were first published in 2004, with a total of 4532 papers published from 2004 to 2021. During this period, there were 185,190 citations, and the citation frequency per paper was 40.87. Overall, research on NETosis has grown annually and can be broadly divided into 3 stages. The first stage (2004–2011) was the slow development phase of NETosis research, with 170 papers and 3536 citations published in this phase, accounting for 3.8% and 1.9% of the total number of publications and citations, respectively. Between 2012 and 2018, there was a stable development stage, with 2031 papers and 69,094 citations published in this phase, accounting for 44.8% and 37.3% of total publications and citations, respectively. Two thousand nineteen is the rapid development stage of NETosis research. From 2019 to 2021, there were 2331 papers and 112,560 citations published in 3 years, accounting for 51.4% and 60.8% of the total publication and citation volume, respectively.

### 3.2. Countries/regions and institutions statistics

**Figure 2.**
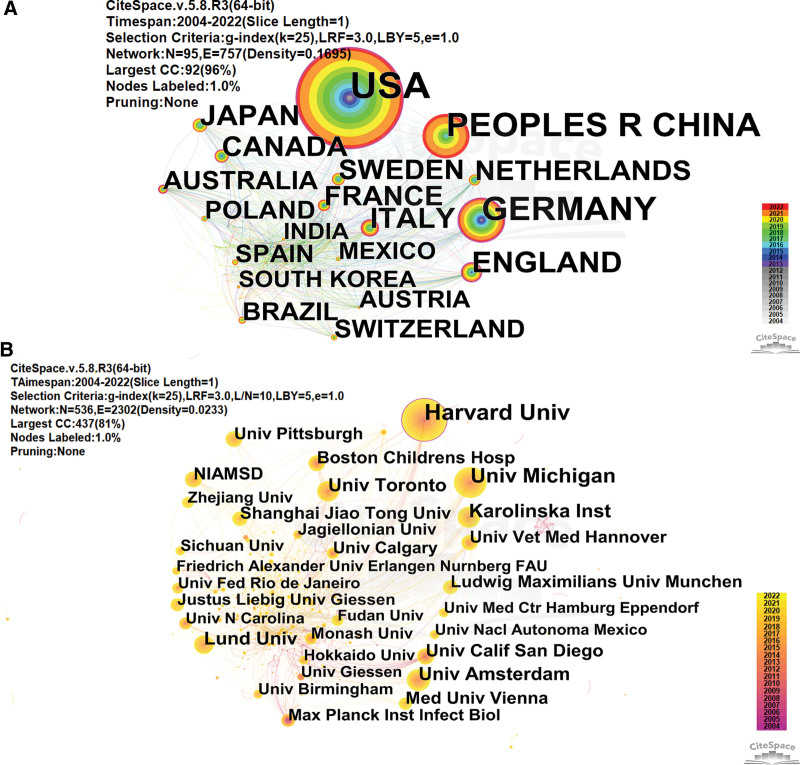
CiteSpace visualization map of countries/regions and institutions on NETosis. (A) Cooperation map of countries/regions in the field of NETosis (N represents the research project in the upper left corner of the network parameter, while E represents collaboration between projects. The nodes represent countries; the larger the node, the more publications. The node consists of numerous annual rings, whose thickness is proportionate to the publications, and the colors indicate the year of publication). (B) Cooperation map of institutions on NETosis. for each year signaled above and below the respective graph).

This study evaluated the countries and institutions that are influential in the field of NETosis through certain indicators, such as publications, citations, and centrality, and consolidation of secondary to primary units in institution analysis. From Figure 2A and B, NETosis-related literature was mainly from 95 countries/regions and 536 institutions. The network density values were small, showing fragmentation of cooperation both between countries and between regions, and their academic communication and cooperation needed to be strengthened. In the country visualization map, the United States, Germany, England, Italy, and Canada nodes had purple circles (BC > 0.1) in their outermost chronologies (Fig. 2A), indicating that these countries were hubs for communication with other countries, and similarly, Harvard University had the same significance in the institutional visualization map (Fig. 2B).

As demonstrated in Table 1, the United States made the most important contribution with 1518 articles (31.17% of 4870 publications), followed by Germany (14.17%, with 690 papers) and China (14.02%, with 683 papers). The United States, Germany, and China accounted for 59.36% of the total publications, far exceeding other countries. The top 10 countries by the number of articles also included England (6.76%, with 329 papers), Italy (5.63%, with 274 papers), Japan (5.40%, with 263 papers), Canada (4.97%, with 242 papers), Sweden (4.5%, with 219 papers), France (4.48%, with 218 papers), and the Netherlands (4.48%, with 218 papers). The first 3 countries by average citation were Canada (84.86), Germany (74.01), and England (65.88). The H-index indicates the number of Np articles published by a journal, author, or nation that have been cited at least h times.^[22]^ Countries that had a high H-index included the United States (132), Germany (104), England (67), and Canada (67). As illustrated in Table 2, 7 of the 10 most published institutions came from the United States. Among them, Harvard University emerged as the leading institution in terms of both publications and centrality in the NETosis field. Its average citation was 103.65, which was slightly lower than the University of California San Diego (118.78) and the University of Michigan (109.34), making them the most influential universities in the field. Based on the combined assessment of publications, average citation number, and centrality, Harvard University (195 papers, 103.65 average citations, 0.18 of centrality), the University of Michigan (117 papers, 109.34 average citations, 0.08 of centrality), Karolinska Institute (83 papers, 91.23 average citations, 0.08 of centrality) and University of California San Diego (54 papers, 118.78 average citations, 0.09 of centrality) were the institutions that contribute the most to the field of NETosis.

**Table 1:** Top 10 productive countries/regions in the field of NETosis.

Rank	Country/region	Quantity	Percentage (N/4870)	Total citations	Average citations	H-index	Centrality
1	USA	1518	31.17	93,372	61.50	132	0.22
2	Germany	690	14.17	51,068	74.01	104	0.25
3	China	683	14.02	16,126	23.61	51	0.02
4	England	329	6.76	21,675	65.88	67	0.27
5	Italy	274	5.63	13,894	50.71	55	0.10
6	Japan	263	5.40	10,152	38.60	44	0.04
7	Canada	242	4.97	20,535	84.86	67	0.11
8	Sweden	219	4.50	13,838	63.19	55	0.08
9	France	218	4.48	14,208	65.17	51	0.03
10	Netherlands	218	4.48	12,466	57.18	55	0.07

**Table 2:** Top 10 institutions performing the studies on NETosis.

Rank	Institution	Country	Quantity	Total citations	Average citations	Centrality
1	Harvard Univ	USA	195	20,211	103.65	0.18
2	Univ Michigan	USA	117	12,793	109.34	0.08
3	Karolinska Inst	USA	83	7572	91.23	0.08
4	Lund Univ	Sweden	68	2699	39.69	0.04
5	Univ Toronto	Canada	67	5307	79.20	0.01
6	Univ Amsterdam	Netherlands	67	5143	76.76	0.05
7	Univ Calif San Diego	USA	54	6414	118.78	0.09
8	Boston Children’s Hosp	USA	51	5145	100.88	0.02
9	NIAMSD	USA	51	3710	72.75	0.03
10	Med Univ Vienna	USA	49	1586	32.37	0.01

### 3.3. Authors analysis

**Figure 3.**
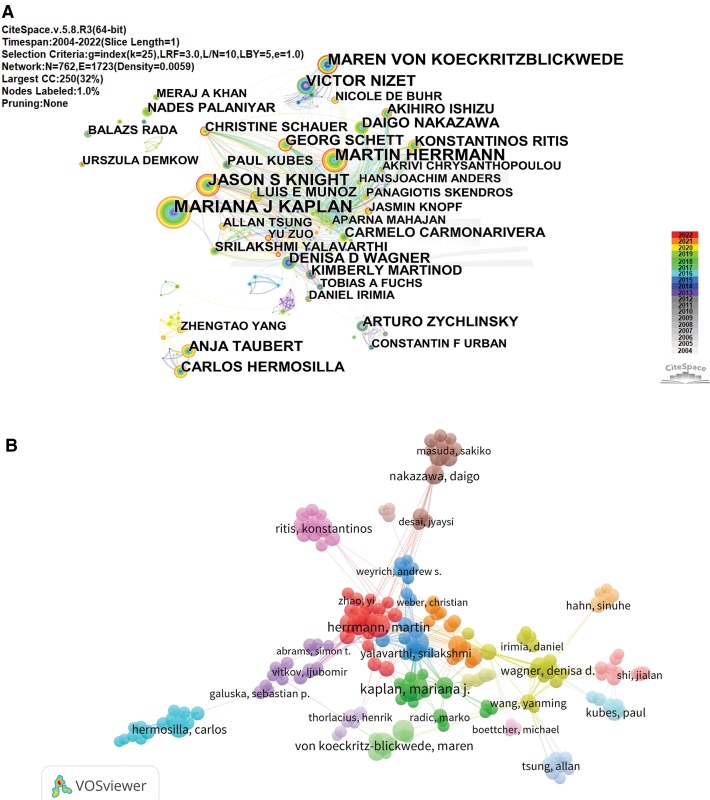
CiteSpace visualization map of authors on NETosis.

Figure 3A shows that 762 major authors were devoted to NETosis research, the network density was just 0.059, which indicated less author collaboration. From Table 3, Mariana J. Kaplan had the most publications (66 publications), followed by Martin Herrmann (49 publications) and Jason S. Knight (46 publications). The first 10 most prolific authors included 4 from the United States, 4 from Germany, 1 from Japan, 1 from Greece, and 1 from England. The visualization map of the authors revealed 5 main author groups (Fig. 3B), with Martin Herrmann, Ritis Konstantinos, Nakazawa Daigo, Wagner Denisa D, and Hermosilla Carlos being the core members of each group. Among them, Martin Herrmann group collaborates closely with one another and communicates frequently with other groups. The top 10 co-cited authors are shown in Table 3. The highest co-citation intensity had Brinkmann V (2960 co-citations), followed by Fuchs TA (1895 co-citations), Papayannopoulos V (1372 co-citations), Clark SR (841 co-citations), Urban CF (818 co-citations), Hakkim A (780 co-citations), Kessenbrock K (648 co-citations), Yipp BG (644 co-citations), Wang YM (639 co-citations), and Li PX (557 co-citations), of which 4 were from the USA, 3 were from Germany, 2 were from Canada, and 1 was from England.

**Table 3:** Top 10 authors and co-cited authors on NETosis.

Rank	Author	Count	Country	Co-cited author	Co-citation	Country
1	Mariana J Kaplan	66	USA	Brinkmann V	2960	Germany
2	Martin Herrmann	49	Germany	Fuchs TA	1895	Germany
3	Jason S Knight	46	USA	Papayannopoulos V	1372	England
4	Maren Von Koeckritzblickwede	40	Germany	Clark SR	841	Canada
5	Victor Nizet	36	USA	Urban CF	818	Germany
6	Anja Taubert	29	Germany	Hakkim A	780	Germany
7	Denisa D Wagner	28	USA	Kessenbrock K	648	USA
8	Carlos Hermosilla	28	Germany	Yipp BG	644	Canada
9	Daigo Nakazawa	27	Japan	Wang YM	639	USA
10	Konstantinos Ritis	27	Greece	Li PX	557	USA
11	Georg Schett	27	England			

### 3.4. Journals analysis

The journal impact factor (IF) and quartiles in Table 4 refer to the 2021 Journal Citation Reports. Journals that were in both the top 10 publications and top 10 citations included Frontiers in Immunology (IF 8.786, Q1), Journal of Immunology (IF 5.446, Q2), PLoS One (IF 3.752, Q2), and Blood (IF 25.476, Q1). Among them, Frontiers in Immunology was both the most published (388 articles) and the most cited journal (10,668 citations) in total, far ahead of other journals. Science (IF 63.798, Q1) was the journal with the highest citation per article (876.88). followed by the Journal of Cell Biology (IF 8.077, Q1) with 656.29 citations per article and Nature Medicine (IF 87.241, Q1) with 550.46 citations per article.

**Table 4:** Top 10 journals in terms of publications and citations.

Rank	Journal	Publications	IF (2021)	JIF quartile	Journal	Total citation	Average citation	IF (2021)	JIF quartile
1	*Front Immunol*	388	8.786	Q1	*Front Immunol*	10,668	27.49	8.786	Q1
2	*Int J Mol Sci*	131	6.208	Q1	*Blood*	7329	122.15	25.476	Q1
3	*PLoS One*	110	3.752	Q2	*Nat Med*	7156	550.46	87.241	Q1
4	*Sci Rep-UK*	107	4.996	Q1	*Science*	7015	876.88	63.798	Q1
5	*J Immunol*	76	5.446	Q2	*J Immunol*	6223	81.88	5.446	Q2
6	*J Leukocyte Biol*	64	6.011	Q2	*PLoS One*	4929	44.81	3.752	Q2
7	*Cell*	62	66.850	Q1	*P Natl Acad Sci USA*	4831	185.81	12.779	Q1
8	*Blood*	60	25.476	Q1	*Cell Death Differ*	4774	341	12.077	Q1
9	*J Thromb Haemost*	48	16.036	Q1	*J Cell Biol*	4594	656.29	8.077	Q1
10	*Thromb Haemostasis*	48	6.681	Q1	*J Exp Med*	4529	215.67	17.579	Q1

IF = impact factor.

**Figure 4.**
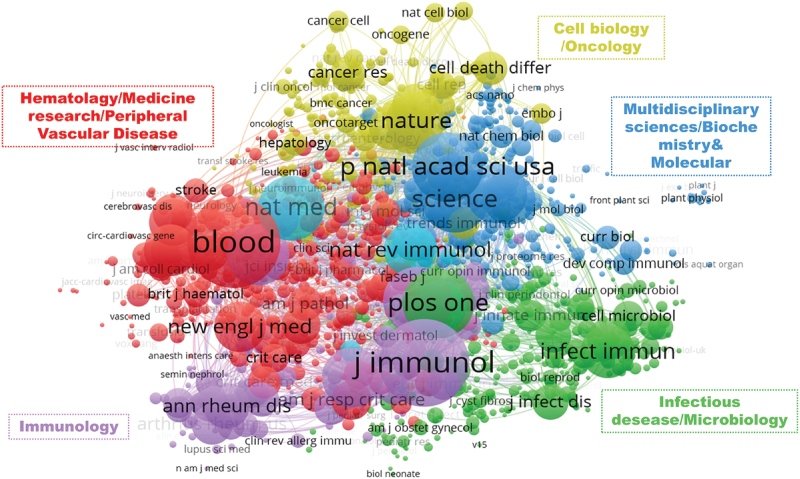
VOSviewer visualization map of co-cited journals on NETosis.

The journal co-citation was first presented by McCain.^[23]^ Co-citation relationship between 2 journals when they are cited in 1 or more publications at the same time.^[24]^ Figure 4 shows a visual network map on NETosis, where the 1510 journals with total co-citations of ≥ 20 are presented in the network map, which mainly includes 5 clusters. The categories of journals with the same color cluster were manually summarized as follows: “Hematology, Medicine Research, Peripheral Vascular Disease” (red cluster), “Immunology” (purple cluster), “Infectious Disease, Microbiology” (green cluster), “Multidisciplinary Sciences, Biochemistry & Molecular” (blue cluster), and “Cell Biology, Oncology” (yellow cluster), indicating that the main subject categories related to NETosis were Hematology, Immunology, Infection, Molecular Biology, Cellular and Cancer. Among them, Blood was the highest co-cited journal, with the Journal of Immunology and P Natl Acad Sci USA following.

**Figure 5.**
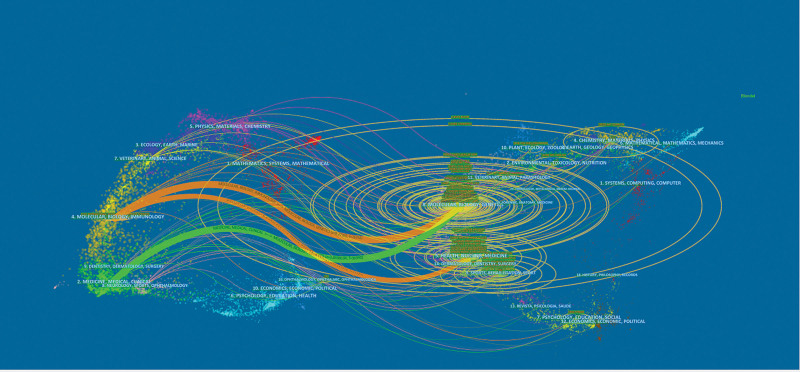
The dual map of journals on NETosis (The size of the ellipse corresponds to the number of publications and authors in NETosis. The vertical axis of the ellipse indicates the number of articles, while the horizontal axis indicates the number of authors. The lines represent citation relationships between journals, and the citation direction is from the cited journal on the right to the citing journal on the left).

The dual map was mainly used to explore the relationship between disciplines. The journal on the left cites the journal on the right. A citation connection was displayed in the curve style as a spline curve that ran from the citing journal’s source to its target.^[25]^ Figure 5 depicts 2 primary colored citation curves. The orange path suggested that articles published in Molecular/Biology/Immunology journals often cited articles from Molecular/Biology/Genetics and Health/Nursing/Medicine journals. Additionally, the green path revealed that articles published in Medicine/Medical/Clinical journals frequently cited articles from Molecular/Biology/Genetics journals. This indicated that basic research on NETosis continues to evolve and was the cornerstone of clinical application research.

### 3.5. Reference analysis

**Figure 6.**
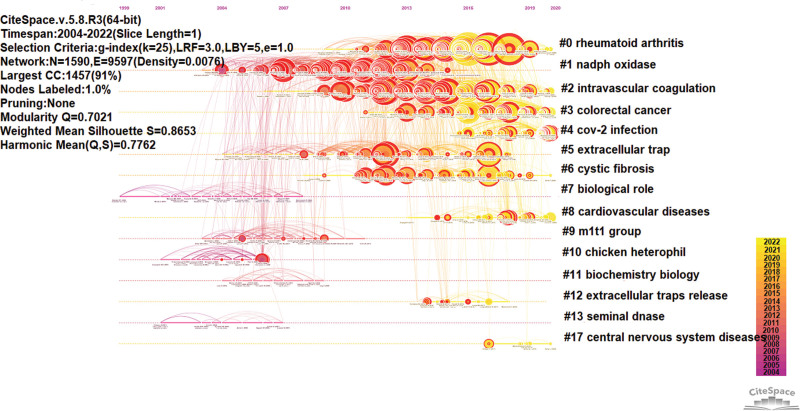
A timeline view of co-citation references on NETosis (Papers with the same clusters are displayed on the same horizontal line, the document’s time is displayed at the top of the view; Clusters are numbered in descending size order, where the more members the cluster contains, the smaller the cluster number).

Small and Marshakova proposed co-citation analysis for the first time in 1973.^[26]^ A co-citation relationship exists when 2 or more papers are cited simultaneously by 1 or more subsequent papers. From Table 5, The top reference with the most co-citations was Papayannopoulos V et al^[27]^ (2018, 544 co-citations), published in Nature Reviews Immunology, with the articles published by Jorch SK et al^[28]^ (2017, 348 co-citations) in Natural Medicine and Lood C et al^[29]^ (2016, 303 co-citations) in Natural Medicine following. Using the CiteSpace clustering tag addition technique, which extracts noun terms from the title, keywords, or abstract of the cited paper to name the literature co-cited network clusters, the data extracted from WoSCC on NETosis were divided into 15 clusters, which were #0 (rheumatoid arthritis), #1 (NADPH oxidase), #2 (intravascular coagulation), #3 (colorectal cancer), #4 (Cov-2 infection), #5 (extracellular trap), #6 (cystic fibrosis), #7 (biological role), #8 (cardiovascular disease), #9 (m1t1 group), #10 (chicken heterophil), #11 (biochemistry biology), #12 (extracellular traps release), #14 (seminal DNase), and #17 (central nervous system disease) (Fig. 6). Among the top 10 co-cited references, 7 papers were articles, 3 were reviews, and 5 papers fell into cluster #0.

**Table 5:** Top 10 co-cited references on NETosis.

Rank	Reference	Type	Co-citations	In cluster
1	Papayannopoulos V, 2018, NAT REV IMMUNOL, V18, P134, DOI 10.1038/nri.2017.105	Review	544	#0
2	Jorch SK, 2017, NAT MED, V23, P279, DOI 10.1038/nm.4294	Review	348	#0
3	Lood C, 2016, NAT MED, V22, P146, DOI 10.1038/nm.4027	Article	303	#0
4	Kenny EF, 2017, ELIFE, V6, P0	Article	239	#0
5	Warnatsch A, 2015, SCIENCE, V349, P316, DOI 10.1126/science.aaa8064	Article	239	#2
6	Brinkmann V, 2012, J CELL BIOL, V198, P773, DOI 10.1083/jcb.201203170	Review	238	#1
7	Zuo Y, 2020, JCI INSIGHT, V5, P0, DOI 10.1172/jci.insight.138999	Article	215	#4
8	Caudrillier A, 2012, J CLIN INVEST, V122, P2661, DOI 10.1172/JCI61303	Article	208	#2
9	von Bruhl ML, 2012, J EXP MED, V209, P819, DOI 10.1084/jem.20112322	Article	207	#2
10	Khandpur R, 2013, SCI TRANSL MED, V5, P0, DOI 10.1126/scitranslmed.3005580	Article	206	#0

The timeline view of reference co-citation can show the evolution of major NETosis research fields over time. Documents in the same linear horizontal position belong to the same cluster. Cluster labels and names are shown on the right side. Clusters are numbered in descending size order, where the more members the cluster contains, the smaller the cluster number. As illustrated in Figure 6, cluster #0 had the most publications, suggesting that the role of NETosis in rheumatoid arthritis is the major research interests in this field. Clusters #0, #2, #3, #4, #8, and #17 had current publications in the last 2 years, indicating that these research directions are still the focus of future studies.

**Figure 7.**
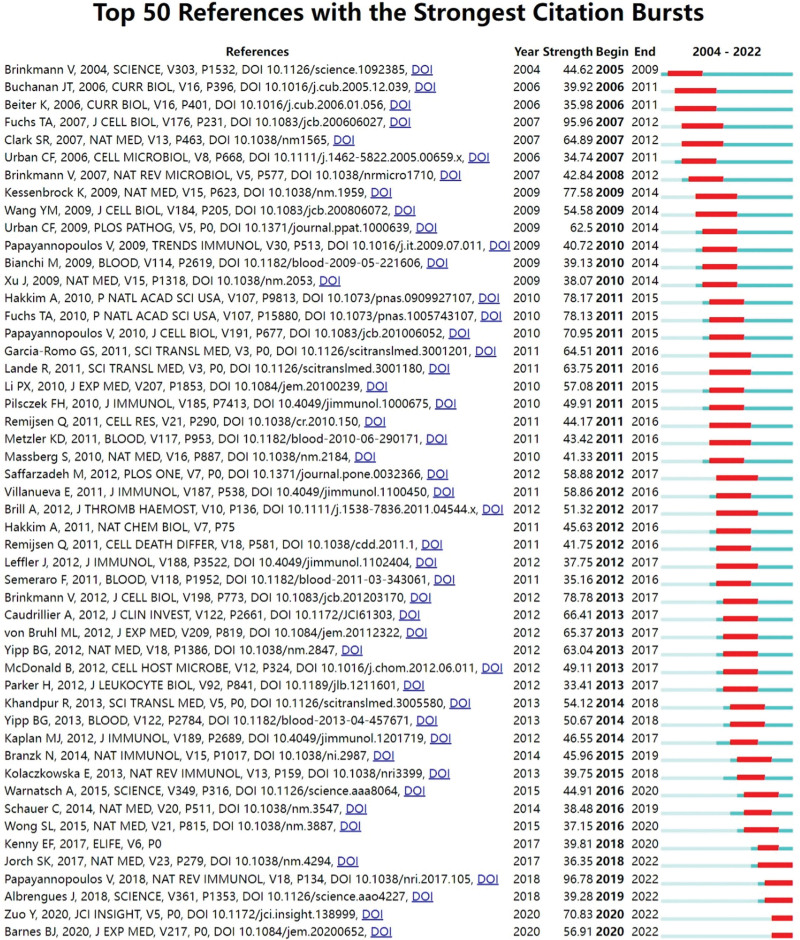
Top 50 references with the strongest citation bursts (Year represents the year the document was published, strength represents the intensity of the burst, begin represents the year the burst began, and end represents the year the burst ended).

From Figure 7, Papayannopoulos V et al (2018, 96.78)^[27]^ published the article with the highest burst intensity. References with strong citation bursts during 2020 to 2022 included Jorch SK et al^[28]^ (2017), Papayannopoulos V et al (2018),^[27]^ Albrengues J et al (2018),^[30]^ Zuo Y et al (2020)^[31]^ and Bames BJ et al (2020),^[32]^ where Jorch SK et al (2017)^[28]^ and Papayannopoulos V et al (2018) discussed the NETosis biology concepts and its action concerning immune protection, coagulation, inflammation, and autoimmune diseases and cancer. ^[27]^ Albrengues J et al (2018)^[30]^ focused on the role of NETosis in cancer metastasis. Zuo Y et al (2020)^[31]^ and Bames BJ et al (2020)^[32]^ both discussed the role of NETosis in COVID-19, indicating that the role and mechanisms of NETosis in cancer and COVID-19 are frontier hotspots for research.

### 3.6. Keywords analysis

Table 6 demonstrates the top 15 keywords after artificially removing some auxiliary words, such as expression, mechanism, release, and disease. The most frequently occurring keyword was a neutrophil extracellular trap (1608), followed by inflammation (485), NETosis (321), extracellular trap (307), infection (261), DNA (261), myeloperoxidase (248), immunity (235), innate immunity (223), systemic lupus erythematosus (221), NADPH oxidase (195), elastase (174), cell death (172), apoptosis (171), and mitochondrial DNA (165). “neutrophil extracellular trap,” “NETosis,” and “extracellular trap” are different expressions of NETs. “DNA,” “myeloperoxidase,” “elastase,” “NADPH oxidase,” and “mitochondrial DNA” are related to the formation of NETs. “Immunity,” “innate immunity,” and “systemic lupus erythematosus” can be attributed to clinical applications, indicating that NETosis plays an important role in innate immunity and autoimmune diseases.

**Table 6:** Top 15 keywords on NETosis.

Rank	Keyword	Count	Centrality
1	Neutrophil extracellular trap	1608	0.07
2	Inflammation	485	0.02
3	NETosis	321	0
4	Extracellular trap	307	0.02
5	Infection	261	0.03
6	DNA	261	0.02
7	Myeloperoxidase	248	0.01
8	Immunity	235	0.01
9	Innate immunity	223	0.01
10	Systemic lupus erythematosus	221	0.02
11	NADPH oxidase	195	0.04
12	Elastase	174	0.01
13	Cell death	172	0.01
14	Apoptosis	171	0.01
15	Mitochondrial DNA	165	0.01

keywords with the strongest burst can explore the research frontiers in the NETosis field. Table 7 lists the keywords with high burst values in the last 3 years, which were ranked in descending order according to their burst values as coronavirus (8.87), ACE2 (7.34), SARS coronavirus (6.69), circulating tumor cell (6.48), pneumonia (5.5), cytokine storm (5.2), citrullination (5.13), prevalence (4.89), neutrophil to lymphocyte ratio (4.83), and cancer (4.61). “Coronavirus,” “ACE2,” “SARS coronavirus,” “pneumonia,” “cytokine storm,” and “neutrophil to lymphocyte ratio” are all related to COVID-19, while “circulating tumor cell,” “prevalence,” and “cancer” are all associated with cancer, indicating that the role and mechanism of NETosis in COVID-19 and cancer are at the forefront of research in this field, which is accordance with the results of co-citation reference analysis.

**Table 7:** Keywords with the strongest burst during 2020–2022.

Rank	Keyword	Strength	Duration
1	Coronavirus	8.87	2020–2022
2	ACE2	7.34	2020-2022
3	SARS coronavirus	6.69	2020–2022
4	Circulating tumor cell	6.48	2020–2022
5	Pneumonia	5.5	2020–2022
6	Cytokine storm	5.2	2020–2022
7	Citrullination	5.13	2020–2022
8	Prevalence	4.89	2020–2022
9	Neutrophil to lymphocyte ratio	4.83	2020–2022
10	Cancer	4.61	2020–2022

## 4. Discussion

Neutrophils, in response to specific stimuli, release line-like structures composed of chromatin and bactericidal proteins and DNA, named neutrophil extracellular traps (NETs), a process called NETosis, since its discovery, it has attracted considerable interest from researchers. This study uses bibliometric software to make a qualitative and quantitative analysis of the abundant literature in the field of NETosis to provide a macro perspective on the current development and future research hotspots in the field of NETosis. We found that the development of the NETosis field is characterized by the following: The research in the field of NETosis consists of 3 main phases, which can be broadly outlined as slow development phase, stable growth phase, and rapid growth phase, the Research on NETosis will continue to flourish in the Future; The United States and Harvard University get the most academic influence, Mariana J Kaplan and Brinkmann V are the most prolific and co-cited authors, respectively, but the scientific research power shows an uneven distribution, with developed countries have higher academic achievements; Frontiers in Immunology is the most prolific journal in terms of publications and citations, Additionally, high-impact journals also include Journal of Immunology, Plos One, Blood, Science, Journal of Cell Biology, and Nature Medicine, these journals either have a high publication volume or high-impact factor; The mechanism of NETosis and its role in innate immunity, autoimmune diseases, particularly systemic lupus erythematosus (SLE) and rheumatoid arthritis, and thrombosis are the focal points of research. The role of NETosis in COVID-19 and cancer metastasis is a hot topic for future research. The definition of NETosis is controversial.

The number of articles and citations of research can reveal the overall situation of scholars’ research on the topic of NETosis. In terms of the annual distribution of the reference published, the number of references and citations shows a trend of increasing year by year, mainly divided into 3 stages. The first 1 was the slow growth stage during 2004 to 2011, in which the research content mainly included the formation and release mechanism of NETs and their role in innate immunity; The second stage was the stable growth stage in 2012 to 2018, during which research was mainly focused on the application of NETosis in diseases (mainly autoimmune diseases, cardiovascular diseases, and cancer); After 2019 began a rapid growth stage, during which a large number of studies on the correlation between NETosis and COVID-19 emerged, which might be the reason for the explosive growth of the publications. NETs were initially found in a specific mode of neutrophil death, and this specific neutrophil death process was later named “NETosis.” Brinkmann V^[33]^ and his colleagues were the first to discover that neutrophils not only kill bacteria through phagocytosis and degranulation, but also produce NETs. These NETs serve as a physical barrier that prevents the spread of bacteria. However, their study also revealed that the extracellular histone component of NETs may exacerbate autoimmune diseases. Since their discovery, there has been a growing interest in studying the mechanisms of NETs formation and their impact on innate immunity and autoimmune diseases. Furthermore, recent research has revealed that NETs may also have a significant role in the development of other diseases. Currently, there have been few studies on developing drugs targeting NETosis, as basic and clinical studies mature, more efforts for the research of NETosis-targeting drugs should be made. We predict that future research on NETosis will continue to grow explosively.

The United States, England, Canada, and Germany are the leading countries in academic output and qualitative indexes, such as average citations and H-index. This signifies that they hold the most influence in the academic world, with the United States taking the top spot. The difference between the United States and other nations is substantial. On a macro level, the strength of a country’s economy is often correlated with its capacity for research. Developed countries tend to have more resources available for scientific inquiry. The United States, for example, is the largest contributor to scientific research in numerous fields. This may be attributed to a number of factors, including its unique federal government system, substantial research funding, and well-organized search system, according to a study on National Institutes of Health funding trends, there was a notable 16.1% increase in the National Institutes of Health budget from 2011 to 2020, the departments that received the highest funding were internal medicine, psychiatry, pediatrics, neurology, and microbiology/immunology/virology^[34]^; Specifically, this is attributable to reputable research institutions and influential academics. Based on the combined assessment of publications, average citation number, and centrality, Harvard University, Univ Michigan, Karolinska Inst, and Univ Calif San Diego are the institutions that contribute the most to the field of NETosis and all 4 institutions are from the United States. Mariana J. Kaplan from the National Institutes of Health in the United States and Brinkmann V from the Max Planck Institute for Infection Biology in Germany are the most prolific and co-cited authors, respectively, this finding is consistent with the bibliometric analysis on NETs in autoimmune diseases.^[35]^ Mariana J. Kaplan focuses primarily on the role of NETosis in autoimmune diseases, Brinkmann V is mainly involved in basic research and mainly focuses on the mechanism of NETosis, where he and his colleagues first discovered that neutrophils can kill bacteria by releasing NETs^.[33]^ There are mainly 5 academic teams, but unfortunately, they collaborate very little, which hinders academic development and innovation. However, Herrmann Martin’s research team stands out as the most collaborative and tackles a diverse range of topics such as inflammation, autoimmune diseases, calculi, acute myocardial infarction, malaria, and COVID-19. This could be attributed to their frequent communication and collaboration with other teams. Such efforts aid in breaking down academic barriers and fostering academic growth. The relationship between a country, its institutions, and its scholars is interconnected. Institutions provide a platform for researchers, and a country’s research status is dependent on high-level institutions and scholars. Conversely, the development of research institutions relies on the state’s support. To promote the long-term growth of the NETosis field, it is beneficial to foster strong collaborations between academic teams, particularly those from different institutions and countries.

Journals play a crucial role in the dissemination and exchange of scientific knowledge. Moreover, the influence of a journal is, to some extent, indicative of the quality of the papers it contains. In this regard, Frontiers in Immunology stands out with 388 publications and 10,668 citations, making it the top journal in terms of both publications and citations. The top 10 publications in journals overlap with their top 10 citations, which include the Journal of Immunology (IF 5.446, Q2), PLoS One (IF 3.752, Q2), and Blood (IF 25.476, Q1), as well as Frontiers in Immunology (IF 8.786, Q1), indicating that these journals not only publish lots of articles but also guarantee the quality of the articles. The highest average citations were found in Science (IF 63.798, Q1), J Cell Biol (IF 8.077, Q1), and Nat Med (IF 87.241, Q1), with 876.88, 656.29, and 550.46, respectively. In summary, Frontiers in Immunology, Journal of Immunology, Plos One, Blood, Science, Journal of Cell Biology, and Nature Medicine are the most influential journals on NETosis, Scholars can choose the journal that best fits their research findings from this list of influential publications. Among them, except for Plos One and the Journal of Immunology, all other journals are classified as Q1. Plos One is a generalist journal with a high publication volume and a short review cycle, which helps attract more researchers to submit manuscripts, and there are many influential studies published there, for example, With a citation burst strength of 58.88 (Fig. 7), Saffarzadeh M et al^[36]^ article “Neutrophil extracellular traps directly induce epithelial and endothelial cell death: a predominant role of histones” in Plos One discovered that histones and myeloperoxidase in NETs can induce host cell toxicity and damage lung tissue.

Keywords are a high-level summary of the topic of the paper, and high-frequency keywords are often taken to identify research hotspots for scientific research. Among the top 15 keywords, “neutrophil extracellular trap,” “NETosis,” and “extracellular trap” are different expressions of NETs. “DNA,” “myeloperoxidase,” “elastase,” “NADPH oxidase,” and “mitochondrial DNA” are related to the formation of NETs. “Immunity,” “innate immunity,” and “systemic lupus erythematosus” can be attributed to clinical applications, indicating that NETosis plays an important role in innate immunity and autoimmune diseases. Combined with the results of reference co-citation analysis, the pathogenesis of NETosis and its effect on innate immune and autoimmune diseases such as systemic lupus erythematosus and rheumatoid arthritis are the hotspots of research in the field of NETosis. Analysis of the keywords with the strongest burst can explore the research frontiers in the NETosis field. Among the top 10 keywords with the strongest burst, “coronavirus,” “ACE2,” “SARS coronavirus,” “pneumonia,” “cytokine storm,” and “neutrophil to lymphocyte ratio” are all related to COVID-19, while “circulating tumor cell,” “prevalence,” and “cancer” are all associated with cancer, indicating that the role and mechanism of NETosis in COVID-19 and cancer are at the forefront of research in this field, which is accordance with the results of co-citation reference analysis.

As research on NETosis progressed, it was discovered that the release of NETs did not always result in the death of neutrophils. In fact, in response to certain stimuli, neutrophils were found to survive after the release of NETs. This type of NETosis was named vital NETosis, while the previous type was referred to as suicide NETosis or lysis NETosis. Desai J^[37]^ considers it necessary to replace the term “NETosis” with a new name, not only for the semantic difference but also to define the molecular mechanism of the drug that targets the release of NETs. Experts recommend using the term “NETosis” only in the context of apparent neutrophil death and the term “NETs formation” in all other cases. In the following, we will focus on describing the hotspots and frontiers.

### 4.1. Hotspots and frontiers

#### 4.1.1. The evolution of NETosis

The basic process of NETosis includes neutrophil activation, chromatin decondensation, collapse and lysis of the granule and nuclear membranes, cytoplasmic and nucleoplasmic component contact, component mixing, cell membrane rupture, and NETs release. In 2004, Brinkmann V and his colleagues^[33]^ published an article in Science in which they first described the formation, structure, and antimicrobial effects of NETs. They found that neutrophils can be activated by IL-8, PMA, or lipopolysaccharide stimulation, and that activated neutrophils produce an extracellular trapping network. The structure and composition of NETs were analyzed by high-resolution scanning electron microscopy and immunofluorescence, respectively. NETs are mainly composed of proteins from granulins (neutrophil elastase (NE), histone G and myeloperoxidase, lactoferrin, and gelatinase), histones (H1, H2A, H2B, H3, and H4), and DNA. NETs disarm pathogens with proteases, such as NE, and exert antibacterial activity through histones. In 2007, The Fuchs TA team^[38]^ found that NETs formation is the final stage in the active cell death program. They showed that depending on reactive oxygen species (ROS) produced by NADPH oxidase, the nucleus loses its shape when neutrophils become activated, Then, EU- and heterochromatin are homogenized, followed by disintegration of the nuclear and granular membranes, with the nuclear membrane decomposing into vesicles and the granular membrane disappearing, and finally, full intermixture of the nucleus, cytoplasm, and granules. The NET is extruded when the cell membrane ruptures. It is also proposed that this process of cell death is unique to apoptosis and necrosis in that there is no DNA fragmentation, it is not dependent on cystatinase, the nuclear membrane rupture is incomplete, and the formation of the NETs requires stimulus activation and is dependent on NADPH oxidase. Steinberg BE and his colleagues^[39]^ referred to this unique and previously unrecognized form of cell death as “NETosis.” The initiating process is the activation of neutrophils by stimulants. Neutrophils that have been activated will initiate protein kinase C and the Raf-MEK-ERK signaling pathway,^[40,41]^ which activate NADPH oxidase to generate ROS. ROS may cause mitochondrial damage and lead to elevated Ca^2+^ levels, thereby triggering Ca^2+^-dependent peptide arginine deiminase 4 (PAD4), which catalyzes histone guanosylation.^[40,42,43]^ Then, by deimination, histone positively charged arginine test chains are converted to polar uncharged guanine side chains, thereby weakening histone binding to DNA,^[44]^ ROS causes NE to dissociate from membrane-associated complexes and activate its protein hydrolytic activity in an myeloperoxidase (MPO)-dependent format, and NE degrades cytoplasmic actin filaments translocating to the nucleus, and cleaving histones to promote chromatin depolymerization.^[45–47]^ Then, gasdermin D is activated by neutrophil protease hydrolysis and can cause nuclear expansion and cytoplasmic membrane rupture^[48,49]^ and nuclear membranes can also be ruptured by cell cycle proteolysis.^[50]^ Then, NETs release occurs. A new mechanism of NETs formation was later discovered that does not depend on oxidants, does not disrupt the plasma membrane, expels nuclear chromatin in a vesicular outgrowth manner, and leaves nucleated cytoplasm for continued microbial uptake.^[38]^ In 2010, Pilsczek FH and his colleagues^[51]^ described this novel NETs release mechanism in a unique response to Staphylococcus aureus that is very rapid (5–60 minutes), does not depend on oxidants, and does not breach the plasma membrane. In this process, after neutrophil activation, the multilobular nuclei quickly become rounded and condensed, then the nuclear membrane is separated inside and outside and vesicles are budded, with both the separated membrane and vesicles filled with nuclear DNA, and the vesicles are extruded to the extracellular space and ruptured, releasing chromatin. This is different from the previously reported mechanism of NETs release, which relies on oxidant-formed NETs taking 3–4 hours to form and the lysis death of neutrophils. This mechanism is now known as vital NETosis.^[52]^ With the discovery of vital NETosis, the traditional lytic NETosis was called suicidal NETosis. The primary distinction between the 2 is that suicide NETosis is characterized by a breakdown of the plasma membrane and a failure of conventional neutrophil function, while vital NETosis has an intact plasma membrane and retains regular functions, such as phagocytosis of live neutrophils. The formation of NETs is complex and depends differently on NADPH oxidase, PAD4, NE, and MPO, depending on the exact activator, and also differs between in vivo and in vitro. For example, nicotine and ionomycin-induced NETosis is non-NADPH oxidase-dependent but mitochondrial ROS-dependent^[53,54]^ and mitochondrial DNA can induce NETosis and is independent of NADPH oxidase through binding to risk-related molecular patterns such as toll-like receptors 9.^[55]^ PMA, Candida albicans, Klebsiella pneumonia, and cholesterol crystals can induce NETosis independently of PAD4.^[56–59]^ PMA-induced NETosis also requires autophagy.^[60]^ NETosis-induced by Pseudomonas aeruginosa is not dependent on MPO,^[61]^ NETosis-induced by ion carriers or salivary mucin occurs in the absence of NE activity.^[62,63]^ Additionally, S. aureus induces vital NETosis.^[64]^

#### 4.1.2. The role of NETosis in innate immunity and autoimmune diseases

NETosis is crucial to innate immunity, and NETs can capture and kill microbes. NETs are fungistatic in their main components (DNA, histones, and calprotectin), whereas calprotectin, a member of the S100A cytoplasmic protein family, inhibits fungal growth by chelating divalent metal ions.^[65,66]^ Granule protein NE degrades bacterial virulence, and NETs immobilize pathogens,^[67]^ limiting their systemic transmission and improving fungicidal efficiency. Clark SR et al^[68]^ found in patients with severe sepsis that plasma also induces tlr4-dependent platelet-neutrophil interactions, leading to enhanced neutrophil activation and NET production, with NETs having the greatest bacterial capture capacity. But some pathogens, such as M1 GAS pneumococci, can express nucleases that degrade NET-associated chromatin and can destroy NETs. The Buchanan JT team^[69]^ has found that mice with necrotizing fasciitis that were subcutaneously injected with the wild-type M1 GAS strain showed greater skin lesions than necrotizing fasciitis mice receiving the identical dose of the M1 DSda1 mutant strain (with significantly reduced DNAase activity), and the wild-type M1 GAS strain could perform a dose-dependent elimination of NETs, whereas the M1 DSda1 mutant was still present in significant amounts of NETs in the companion assays. Beiter K and his colleagues^[70]^ have shown that NETs can trap but not kill pneumococci. Pneumococci express an endonuclease programmed by end A on their surface; end A degrades NETs and allows bacterial escape, allowing bacteria to spread from local sites to the lungs and then to the bloodstream.

The common feature that exists between different autoimmune diseases is the inflammatory response to autoantigens, such as in patients with SLE, where high levels of antinuclear and anti-DNA antibodies are present^[71]^ and anti-neutrophil cytoplasmic antibodies and plasma autoantibodies against guanosine peptides and histones are prevalent in rheumatoid arthritis patients.^[72,73]^ NETosis discharges endogenous cellular components that act as autoantigens, induce host immune responses, and discharge host-associated molecular patterns that augment inflammatory responses, which, in turn, can induce NETosis, leading to the perpetuation of pathological mechanisms. For example, Immune complexes, such as DNA-bacterial peptides released by NETs, are present in the serum of SLE patients and can trigger plasmacytoid dendritic cells to create interferon through toll-like receptors 9, and interferon-α can induce NETosis.^[74]^ Additionally, a subpopulation of neutrophils, low-density neutrophils, has been identified in SLE patients, and low-density neutrophils are more likely to induce NETosis.^[75]^ However, in patients with gout, NETs degrade cytokines and chemokines to attenuate the inflammatory response.^[54,76,77]^

#### 4.1.3. NETosis and thrombosis

Activated platelets induce NETosis, and NETs act as platelet scaffolds and activate FXII, which binds fibrin to red blood cells to form a dense network, while the NE of NETs and histone G degrade coagulation inhibitors.^[78,79]^ Thrombosis is also a mechanism by which NETosis accelerates the progression of atherosclerosis, cancer, and COVID-19. However, thrombogenesis is also considered a means of immune defense, and NETosis can be induced by blood-borne pathogens. NETosis induces thrombosis in the microvasculature, and NETs components kill bacteria and limit their dissemination. NETs can damage endothelial cells, causing endothelial dysfunction and attracting monocytes, which can differentiate into macrophages and form foam cells after phagocytosis of large amounts of lipoproteins and cholesterol, gradually forming atherosclerotic plaques. Advanced plaques exposed to neutrophil-derived proteases and ROS lead to plaque instability.^[80,81]^ The Maugeri N team^[82]^ has found that coronary thrombus mainly consists of activated platelets, neutrophils, and NETs near the platelets. Activated platelets deliver high mobility group box 1 protein to neutrophils, allowing them to participate in autophagy and NET formation. The interaction of thrombin-activated platelets and polymorphonuclear leukocytes at the site of plaque rupture, according to Stakos DA and his colleagues,^[83]^ leads to the formation of local NETs and the delivery of active tissue factors in acute ST-segment elevation myocardial infarction.

#### 4.1.4. NETosis and cancer

Cancer cells can release chemokines to attract neutrophils and induce NETosis.^[84,85]^ NETs have a crucial role in tumor recurrence and dissemination. Matrix metallopeptidase 9 and NE in NETs break down extracellular matrix laminin, this triggers the integrin 31/FAK/ERK/MLCK/YAP signaling cascade, which wakes up dormant cancer cells and stimulates the growth and survival of cancer cells.^[30]^ The DNA of NETs interacts with the transmembrane protein coiled-coil domain-containing protein 25, and coiled-coil domain-containing protein 25 stimulates the ILK – Parvin pathway, attracting cancer cells to distant metastases.^[86]^ NETs are capable of capturing circulating tumor cells to facilitate cancer dissemination^[87]^; the mechanism could be related to NET-derived IL-8 activating the nuclear factor-κB pathway,^[88]^ NETs can also accelerate the conversion of cancer cells epithelial morphology to a mesenchymal phenotype, which confers mobility and invasion.^[89]^ Additionally, NETs not only provide a physical barrier that prevents pathogen transmission but also contribute to immune evasion by preventing cytotoxic cells from entering growing tumors.^[90]^

#### 4.1.5. NETosis and COVID-19

COVID-19 is due to the severe acute respiratory syndrome coronavirus 2, which enhances pro-inflammatory mediator release from airway epithelial cells and macrophages. Pro-inflammatory mediators, including IL-8, can induce NETosis.^[91]^ severe acute respiratory syndrome coronavirus 2 also activates platelets, which can also induce NETosis.^[92]^ Patients with COVID-19 have a higher number of low-density granulocytes, which can cause NETosis.^[93]^ Zuo Y and his colleagues^[31]^ found higher levels of cytosolic free DNA, MPO-DNA, and guanylate histone H3 in the serum of COVID-19 patients, they also found that serum from COVID-19 patients induced NET release from normal neutrophils in vitro. Barnes BJ^[32]^ reported pulmonary infiltration of neutrophils in COVID-19-related deaths autopsy specimens. The ratio of neutrophils to lymphocytes was found to correlate with the severity of COVID-19,^[94]^ which can produce excess cytokines such as tumor necrosis factor (TNF), IL-1, IL-6, IL-8, IL-12, and IL-17, creating a cytokine storm that can cause vasodilation, impair endothelial function, induce leukocyte recruitment and endothelial cell permeability, cause secondary inflammatory responses, and activate coagulation pathways,^[95,96]^ ultimately leading to multiorgan dysfunction and aggravating the mortality of COVID-19.

### 4.2. Limitations

First, all the documents were obtained from the WoSCC database, and only articles and reviews were considered, which might have led to the omission of some publications. Second, the results obtained would vary slightly with different bibliometric analysis tools or parameter settings, but bibliometrics can help newcomers quickly become familiar with research trends, research hotspots, and research frontiers in the field of NETosis.

## 5. Conclusion

We used CiteSpace and VOSviewer software to analyze 4870 articles in the field of NETosis research. We found that NETosis research is in an exciting time, with a sharp rise in publications of NETosis-related research after 2019, which may be related to the large number of papers on this topic regarding the action of NETosis in COVID-19. The United States, Germany, England, and Canada are the most impactful countries in the field of NETosis, with the United States in the lead and China with more publications but relatively few citations, requiring an improvement of the quality of the literature. Harvard University, University of Michigan, Karolinska Institute, and University of California San Diego are the institutions that have contributed most to the field of NETosis research, with Harvard University as the leader. Mariana J Kaplan is the most prolific author, and Brinkmann V is the most co-cited author. Cooperation and communication should be strengthened between countries, different institutions, and different author teams to better promote the future development of NETosis. Frontiers in Immunology, Journal of Immunology, PLoS One, Blood, Science, Journal of Cell Biology, and Nature Medicine are the most influential journals in the NETosis field.

In this study, we found that the mechanism of NETosis has been the focal point of research in this field, The term “NETosis” should be used cautiously and only when the release of NETs is associated with neutrophil death; otherwise, the term “NETs formation” should be used. In addition, the interplay between NETosis and other modes of programmed cell death has yet to be fully elucidated. Besides, in the field of NETosis, the effect of NETosis on innate immune and autoimmune diseases, particularly SLE and rheumatoid arthritis, is a hot topic. Notably, NETosis-induced thrombosis is the pathological mechanism by which NETosis exacerbates COVID-19, cancer, acute myocardial infarction, and ischemic stroke. Therefore, the role of NETosis in thrombogenesis is also a hot topic in prospective research.

The role of NETosis in COVID-19 and cancer recurrence and metastasis is the research frontier in this field. Future research will focus on the effect of NETosis-induced cytokine storms on COVID-19 organ function impairment and the mechanism by which NETs trap tumor cells to promote metastasis.

## Acknowledgments

We appreciate the CiteSpace and VOSviewer developers for providing software free of charge. This research was funded by the Technology Innovation of the China Academy of Chinese Medical Sciences (CI2021A01407, CI2021A01408, and CI2021A01406), the National Natural Science Foundation of China (no. 81774143), and the Qihuang Project for Inheritance and Innovation of Traditional Chinese Medicine (01012003), China Academy of Chinese Medicine Scientific Foundation (ZZ13-YQ-010).The authors declare that the research was conducted in the absence of any commercial or financial relationships that could be construed as a potential conflict of interest.

## Author contributions

**Conceptualization:** Hongqin Wang, Qingbing Zhou, Ying Zhang.

**Data curation:** Hongqin Wang, Zijun Jia.

**Formal analysis:** Hongqin Wang.

**Funding acquisition:** Fengqin Xu, Ying Zhang.

**Investigation:** Hongqin Wang, Li Liu.

**Methodology:** Hongqin Wang, Yifei Qi, Qingbing Zhou.

**Project administration:** Fengqin Xu, Ying Zhang.

**Resources:** Fengqin Xu.

**Supervision:** Fengqin Xu, Ying Zhang.

**Software:** Hongqin Wang, Xiaolin Liu.

**Writing – original draft:** Hongqin Wang.

**Writing – review & editing:** Hongqin Wang.
